# Implementation Issues of Adaptive Energy Detection in Heterogeneous Wireless Networks

**DOI:** 10.3390/s17040932

**Published:** 2017-04-23

**Authors:** Iker Sobron, Iñaki Eizmendi, Wallace A. Martins, Paulo S. R. Diniz, Juan Luis Ordiales, Manuel Velez

**Affiliations:** 1 University of the Basque Country UPV/EHU, 48013 Bilbao, Spain; inaki.eizmendi@ehu.eus (I.E.); juanluis.ordiales@ehu.eus (J.L.O.); manuel.velez@ehu.eus (M.V.); 2 Federal University of Rio de Janeiro (UFRJ), Rio de Janeiro 21941-972, Brazil; wallace.martins@smt.ufrj.br (W.A.M.); diniz@smt.ufrj.br (P.S.R.D.)

**Keywords:** energy detection, SDR implementation, USRP, cooperative networks

## Abstract

Spectrum sensing (SS) enables the coexistence of non-coordinated heterogeneous wireless systems operating in the same band. Due to its computational simplicity, energy detection (ED) technique has been widespread employed in SS applications; nonetheless, the conventional ED may be unreliable under environmental impairments, justifying the use of ED-based variants. Assessing ED algorithms from theoretical and simulation viewpoints relies on several assumptions and simplifications which, eventually, lead to conclusions that do not necessarily meet the requirements imposed by real propagation environments. This work addresses those problems by dealing with practical implementation issues of adaptive least mean square (LMS)-based ED algorithms. The paper proposes a new adaptive ED algorithm that uses a variable step-size guaranteeing the LMS convergence in time-varying environments. Several implementation guidelines are provided and, additionally, an empirical assessment and validation with a software defined radio-based hardware is carried out. Experimental results show good performance in terms of probabilities of detection ($P_{d}>0.9$) and false alarm ($P_{f}∼0.05$) in a range of low signal-to-noise ratios around [-4,1] dB, in both single-node and cooperative modes. The proposed sensing methodology enables a seamless monitoring of the radio electromagnetic spectrum in order to provide band occupancy information for an efficient usage among several wireless communications systems.

## 1. Introduction

An important bottleneck in current broadband wireless communication systems is spectrum scarcity. Among the proposals to address this issue, the continuous sensing of particular spectrum bands in order to detect the so-called white spaces is quite promising. Users that otherwise would not be able to communicate can leverage this knowledge to opportunistically use the available spectrum portions. The sensing stage here is of paramount importance and, in this context, it is implemented by devices with built-in antenna (or antenna array), down converters, analog-to-digital converters (ADCs), and digital processors that are fed by the resulting digital samples and may perform additional expert processing. The devices capable of performing those tasks are called cognitive radio (CR) nodes; in fact, following an intelligent sensing framework, the main challenges regarding spectrum sensing by CR nodes can be cast within the digital signal processing context, as will be further clarified.

Current wireless communication models state new ecosystems such as 5G in order to integrate different wireless communication solutions into a unified structure that connects people, machines and devices on a massive scale, besides offering a variety of ubiquitous services and applications [1]. In order to favor this model, the shared use of radio spectrum resources is also promoted [2] by providing alternatives for the coexistence of non-coordinated heterogeneous wireless systems operating in the same band. At this point, spectrum sensing is the first stage of the dynamic spectrum access cycle that enables a virtually harmless coexistence. The basic functioning principle of this solution relies on a usually unlicensed or secondary user (SU), capable of observing whether a specific frequency band is being utilized by a primary, or licensed, user (PU). If the frequency band is available, then the secondary user can occupy it without interfering with primary terminals. In addition, if the authorized terminal restarts transmission, the secondary terminal jumps off into a different band, or modifies its transmission scheme, while staying in the same frequency band, in order to minimize interference [3,4,5]. Several wireless technologies such as Zigbee, WiFi and Bluetooth already coexist in unlicensed bands (i.e., Industrial, Scientific and Medical (ISM) bands), employing spectrum sensing as a listen-before-talk strategy in order to minimize mutual interferences in the spectrum sharing scenario. In addition, other wireless schemes such as Long Term Evolution (LTE)-unlicensed extend LTE to unlicensed spectrum by aggregating unlicensed carriers with licensed ones through carrier aggregation or solely operating in unlicensed bands [6], where spectrum sensing will play an important role. Lastly, we can also mention the recently emerged concept named licensed shared access (LSA) [7,8]. LSA is a supervised shared access proposal based on an exclusive regime of spectrum sharing among incumbents—i.e., PUs, which have the right to commercially exploit a given wireless spectrum portion—and LSA licensees—i.e., licensed users that leased an incumbent’s spectrum band, which can then be used when permission is granted. The entity responsible for granting permissions is a denominated LSA controller, whose decisions are taken based on spectrum availability information provided by incumbents to the LSA repositories [9]. In this context, spectrum sensing technologies facilitate the decisions of LSA controllers by providing LSA repositories with dynamic and up-to-date radio-environment maps (REMs) [1,10]. More specifically, this dynamic knowledge and update of REMs is acquired via processing of spectrum measurements collected from intelligent sensors, consisting of measurement-capable devices (MCDs) with geo-location information.

A plethora of spectrum sensing techniques can be found in the literature, where energy detection (ED) plays a key role in low-complexity applications due to its inherent computational simplicity in terms of implementation and no need of prior information about PUs [5,11,12,13,14]. Variants of the ED technique, including adaptive ED solutions, have been proposed in order to address those cases in which the conventional energy detector is unreliable due to environmental circumstances, such as insufficient signal strength, rapid noise-power fluctuations, or background interferences [15,16]. Whenever possible, cooperative strategies are employed to increase the detection reliability in either centralized or distributed manners [17,18]. In this context, distributed least-mean-squares (LMS)-based algorithms have been widely employed for adaptive ED purposes [19,20,21,22,23,24]. Commonplace among those skeptical researchers and practitioner engineers is the fact that most of the works in this area are analyzed from theoretical and simulation viewpoints solely, thus relying on several assumptions and simplifications which, eventually, lead to conclusions that do not necessarily meet the requirements imposed by real propagation environments. Exceptions to this rule include [25,26] and references therein, which consider some practical issues of conventional ED, and [27,28,29], which consider practical opportunistic spectrum access from a cross-layer perspective, analyzing medium access control (MAC) and other layers of the protocol stack.

In this work, we analyze the distributed LMS-based ED technique proposed in [24] from a practical viewpoint. In theoretical works, environment parameters, such as signal-to-noise ratio (SNR) and noise variance, are initially fixed to analyze the performance of the algorithm. Based on those predefined values, the step-size of the LMS algorithm, which controls the convergence speed of the algorithm, is accordingly chosen. In real scenarios, shadowing due to moving objects or people along with other environmental impairments make the environment time-varying. As a consequence, those parameters which are considered constant in theoretical works are no longer time-invariant and must be updated online. These reasons motivated this work to deal with the implementation issues of the previously-proposed adaptive ED algorithm and present a new practical proposal of the theoretical LMS-based ED algorithm. The proposed modifications make the original algorithm practical to work with minimal tuning in heterogeneous wireless scenarios. In addition, an empirical assessment and validation with a software defined radio (SDR)-based hardware implementation is carried out.

Thus, the main contributions of the paper are: (1) a new proposal of a variable step-size (VSS) LMS ED algorithm which computes online the step-size in order to ensure the convergence of the adaptive solution proposed in [24] for time-varying SNR environments; (2) practical solutions for noise variance and SNR estimations as well as data sharing; (3) implementation guidelines of the VSSLMS-based ED algorithm for single-node and cooperative modes; (4) an implementation of the practical VSSLMS-based ED scheme in an SDR-based framework using Universal Software Radio Peripheral (USRP) platforms for both single-node and cooperative ED; and, finally; (5) the evaluation of the sensing system using lab measurements in a real indoor radio-propagation scenario.

The ED-based sensing methodology proposed in this paper pursues an actual seamless monitoring of the radio environment through the received electromagnetic signal strength processing in MCDs (i.e., USRP devices). The ED-based sensing solutions are given for stand-alone or cooperative modes, in which the latter provides a more accurate and reliable monitoring in areas with low SNR. The described sensing procedure can be employed for different purposes such as opportunistic spectrum access in spectrum sharing scenarios or generation of REMs.

The paper is organized as follows. Section 2 briefly reviews the LMS-based ED algorithm proposed in [24]; Section 3 deals with the three main implementation issues of the algorithm, namely noise variance and SNR estimation, variable step-size computation, and cooperative issues; in Section 4, the SDR-based testbench is described. The results corresponding to the lab measurements and further discussions are presented in Section 5, in which transient and steady-state analyses are firstly performed in Section 5.1, while the detection performance for different schemes is analyzed in Section 5.2. Finally, conclusions are drawn in Section 6.

## 2. Review of Adaptive ED Algorithm

Let us consider a cognitive radio network where secondary users spatially distributed perform spectrum sensing in a selected frequency band in order to detect the presence of licensed or primary users. The primary-user signal and the input noise at the secondary-user receiver are assumed random and zero-mean Gaussian distributed with covariance matrices $\Sigma_{s}=\sigma_{s}^{2}I_{N}$ and $\Sigma_{w}=\sigma_{w}^{2}I_{N}$, respectively. Thus, the received signal $x={\left[x_{1},...,x_{N}\right]}^{T}$ can be expressed as

(1) $$\begin{array}{c} x=\beta s+w, \end{array}$$

where $s={\left[s_{1},...,s_{N}\right]}^{T}$ is the PU signal, $w={\left[w_{1},...,w_{N}\right]}^{T}$ denotes the input noise, and β works as a selector of the environment characteristic under the hypotheses $H_{0}$ (absence of PU signal, i.e., β=0) and $H_{1}$ (presence of PU signal, i.e., β=1). The energy detector of the *m*-th secondary user computes a local test statistic $y_{m,k}$ from *N* received samples at the time slot *k* as

(2) $$\begin{array}{c} y_{m,k}={∥x∥}^{2}=\sum_{n=1}^{N}{\left|x_{n}\right|}^{2}. \end{array}$$

The local test statistic is then used to compute an adaptive LMS-based test statistic (cf. [24] for more details), which can then be employed in both cooperative and single-node contexts. As a result, the update equation of the test statistic $\omega_{m,k}$ to be used in a distributed detection process at the *m*-th SU can be expressed as

(3) $$\begin{array}{ccc} \omega_{m,k+1} & = & \omega_{m,k}+\mu_{m}\sum_{i\in N_{m}}c_{i}\epsilon_{i,k}{\tilde{u}}_{i,k}\;, \end{array}$$

where ${\tilde{u}}_{i,k}=\left|y_{i,k}-\gamma_{i}\right|$ represents the adaptive filter input associated with the *i*-th neighboring user within the neighborhood of the *m*-th user, denoted as $N_{m}$. It is worth noticing that (3) is the generalized expression for single-node and distributed cooperative detection. When cooperation is possible, the LMS-based test statistic uses the local test statistics obtained from the *m*-th node and their neighboring SUs, i.e., $y_{i,k}\;\mathrm{for}\;i\in N_{m}$. When single-node operation is chosen, the LMS-based test statistic uses only the test statistic of the *m*-th SU, i.e., $y_{m,k}$ since $\mathrm{card}(N_{m})=1$. The value $\gamma_{i}$ is the threshold over the test statistic $y_{i,k}$ for a predefined probability of false alarm $P_{f}=\mathrm{Pr}\left(y_{i,k}>\gamma_{i}\;|\;H_{0}\right)$. In general, $\gamma_{i}$ can be computed by considering $y_{i,k}$ as being Gaussian distributed, a reasonable approximation for sufficiently high *N* in practice [24,30]. The parameter $\mu_{m}$ represents the step-size of the adaptive algorithm and the output-error coefficient $\epsilon_{i,k}$ is computed as

(4) $$\begin{array}{c} \epsilon_{i,k}={\tilde{d}}_{i,k}-\omega_{m,k}{\tilde{u}}_{i,k}\;, \end{array}$$

where ${\tilde{d}}_{i,k}=d_{i,k}-\gamma_{i}$, with $d_{i,k}$ being the desired signal, which can be computed at each instant *k* as

(5) $$\begin{array}{c} d_{i,k}=\left(1-\alpha \right)y_{i,k}+\alpha d_{i,k-1}\;, \end{array}$$

in which α is a scalar close to but less than 1 (α=0.95 is widely used). Moreover, the coefficients $c_{i}$ must satisfy $\sum_{i\in N_{m}}c_{i}=1$ and are chosen in order to perform uniform or weighted cooperation. When cooperation is uniform, weights are computed as $c_{i}=1/\mathrm{card}\left(N_{m}\right)$. Weighted cooperation is carried out as a function of parameters such as number of linked nodes [31], noise variance, or SNR estimates [24]. The selection of the weighted strategy shall depend on the available shared information from the neighbors at the *m*-th SU.

Following the update process in (3), the detection test is then performed:

(6) $$\omega_{m,k}\overset{H_{1}}{\underset{H_{0}}{⋛}}{\tilde{\gamma }}_{m}\;,$$

where ${\tilde{\gamma }}_{m}$ is the new detection threshold for the neighborhood $N_{m}$. Assuming that the distribution of $\omega_{m,k}$ at steady state can be approximated to a Gaussian distribution [24], we can express the probability of false alarm of the detector for a certain threshold ${\tilde{\gamma }}_{m}$ as

(7) $$P_{f}=\mathrm{Pr}\left(\omega_{m,k}>{\tilde{\gamma }}_{m}\;|\;H_{0}\right)=Q\left(\frac{{\tilde{\gamma }}_{m}-E{\left[\omega_{m,k}\right]}_{H_{0}}}{\sqrt{\mathrm{Var}{\left[\omega_{m,k}\right]}_{H_{0}}}}\right),$$

where $E{\left[\omega_{m,k}\right]}_{H_{0}}$ and $\mathrm{Var}{\left[\omega_{m,k}\right]}_{H_{0}}$ are the expectation and variance of $\omega_{m,k}$, respectively, when hypothesis $H_{0}$ holds. The derivations of those values can be found in [24]. The *Q*-function is defined as $Q\left(z\right)=\int_{z}^{\infty }\frac{1}{\sqrt{2\pi }}e^{-\frac{x^{2}}{2}}dx$.

Consequently, from (7), the threshold ${\tilde{\gamma }}_{m}$ can be obtained for a predefined $P_{f}$ as

(8) $${\tilde{\gamma }}_{m}=E{\left[\omega_{m,k}\right]}_{H_{0}}+Q^{-1}\left(P_{f}\right)\sqrt{\mathrm{Var}{\left[\omega_{m,k}\right]}_{H_{0}}}\;.$$

## 3. Implementation Issues of Adaptive ED

This section deals with the main practical issues arising from the implementation of the previously described algorithm. In real applications, the parameters which are usually assumed known or computed offline in theoretical works need to be updated online to make the algorithm practical and self-adjustable. In this sense, we present a series of pragmatical solutions which enable the actual implementation of the adaptive ED algorithm described in the previous section in an SDR-based platform. Firstly, a practical method for the noise variance $\sigma_{m}^{2}$ and SNR estimation is presented. Secondly, we will propose a new version of the LMS-based ED algorithm based on a variable step-size strategy in order to ensure the convergence of the algorithm in time-varying environments. In addition, the behavior of the VSS proposal is analyzed in the transient states. Finally, some aspects of data sharing in cooperative detection are addressed along with a general view of the new practical proposal.

### 3.1. Noise Variance and SNR Estimation

In practical scenarios, it is necessary to estimate the noise variance in order to compute the thresholds γ and $\tilde{\gamma }$ (sometimes, we shall omit the index that identifies the SU node (e.g., *m*) for the sake of notation simplicity). Such a task can be conducted before running the adaptive algorithm by considering that $y_{m,k}$ is drawn from a Gaussian distribution whose parameters depend on the particular hypothesis. More specifically, $y_{m,k}∼N\left(N\sigma_{m}^{2},2N\sigma_{m}^{4}\right)$ if $H_{0}$ holds, whereas $y_{m,k}∼N\left(\left[N+N\eta_{m,k}\right]\sigma_{m}^{2},2\left[N+2N\eta_{m,k}\right]\sigma_{m}^{4}\right)$ if $H_{1}$ holds. As $d_{m,k}\approx E\left[y_{m,k}\right]$ in the steady state, it immediately follows that $d_{m,k}\approx E{\left[y_{m,k}\right]}_{H_{1}}$ when $H_{1}$ holds and $d_{m,k}\approx E{\left[y_{m,k}\right]}_{H_{0}}$ when $H_{0}$. Hence, one can estimate the SNR during PU’s transmissions as:

(9) $$\begin{array}{c} {\hat{\eta }}_{m,k}=\frac{d_{m,k}}{N\sigma_{m}^{2}}-1. \end{array}$$

When the channel is idle, noise variance can be estimated from $d_{m,k}$ since $d_{m,k}\approx E{\left[y_{m,k}\right]}_{H_{0}}=N\sigma_{m}^{2}$ after some iterations, while ${\hat{\eta }}_{m,k}$ in (9) will tend to zero. The SNR estimates can be used to gauge the strength of the signal and decide the cooperation strategy when neighbors are available. In addition, estimated values ${\hat{\eta }}_{m,k}$ can be used for the proposed SNR-based weighted cooperative method proposed in [24].

### 3.2. A Variable Step-Size LMS-Based ED Proposal

One of the main challenges associated with the use of LMS algorithms is the proper choice of their step-size parameter. When the step-size is chosen close to its maximum value (that guarantees convergence), the algorithm converges rapidly but the resultant error floor is high. In contrast, reducing the step-size enhances the error performance, but the transient state of the algorithm becomes longer. In order to overcome this effect, variable step-size (VSS) strategies have been suggested in the literature for different purposes [32,33,34]. The many possible choices for the adaptation of the step-size come from the multitude of scenarios where adaptive algorithms can be applied [32]. We shall analyze in this section the time-varying ED problem in order to propose a balanced VSS strategy in terms of stability and performance.

Convergence of the parameter $\omega_{m,k}$ in (3) is ensured when $0<\mu_{m}<\frac{1}{E\left[{\tilde{u}}_{m,k}^{2}\right]}$. As the adaptive filter input can fluctuate between two states, namely presence and absence of signal, a good choice of $\mu_{m}$ can be performed following the criterion given by:

(10) $$0<\mu_{m}<\min\left\{E{\left[{\tilde{u}}_{m,k}^{2}\right]}_{H0}^{-1},E{\left[{\tilde{u}}_{m,k}^{2}\right]}_{H1}^{-1}\right\}.$$

**Figure 1:**
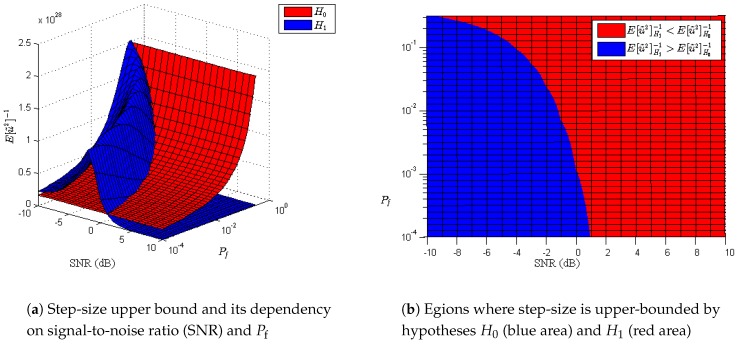
Step-size upper bound analysis.

In Figure 1, the values of $E{\left[{\tilde{u}}_{m,k}^{2}\right]}^{-1}$ for hypotheses $H_{0}$ and $H_{1}$ are depicted for SNRs within the interval [-10,10] dB and $P_{f}$ within the interval $\left[10^{-4},10^{-0.5}\right]$, considering the measured value of noise variance as $\sigma_{w}^{2}=5.6\times 10^{-16}$. One can observe that the maximum limit of the step-size is given by the statistics of hypothesis $H_{0}$ when SNR is negative and the probability of false alarm is low. However, when SNR increases, $E\left[{\tilde{u}}_{m,k}^{2}\right]$ under $H_{1}$ is dominant, and, consequently, the step-size upper bound is given by $E{\left[{\tilde{u}}_{m,k}^{2}\right]}_{H_{1}}^{-1}$. As the sensed environment is usually time-varying due to ubiquitous shadowing effects, SNR can fluctuate during the sensing, eventually modifying the convergence criterion of the algorithm. In this context, the use of an adaptive strategy to compute $\mu_{m}$ allows for dealing with this time-varying effect.

As mentioned before, several VSS algorithms have been previously proposed to increase the speed of convergence or to reduce the mean square error depending on the particular application. In detection problems, one cannot usually estimate offline the noise variance of the equipment, for we do not know a priori which hypothesis holds. As we have seen in Figure 1, the step-size upper bound may depend on $H_{1}$ statistics in some particular scenarios. For that reason, we need an adaptive strategy to compute a step-size satisfying the convergence criterion. In order to do that, we propose computing the following ancillary parameters for each $i\in N_{m}$:

(11) $$\begin{array}{c} \left\{\begin{array}{c} p_{i,k}=\left(1-\alpha \right){\tilde{u}}_{i,k}^{2}+\alpha p_{i,k-1}\\ {\bar{\mu }}_{i,k}=\min\left\{{\bar{\mu }}_{i,k-1}\;,\;2^{-\left\lceil \log_{2}\left(p_{i,k}\right)\right\rceil }\right\} \end{array}\right. \end{array}$$

and then choose the step-size as

(12) $$\mu_{m,k}=\min_{i\in N_{m}}\left\{{\bar{\mu }}_{i,k}\right\}.$$

At this point, it is worth highlighting that the threshold $\tilde{\gamma }$ in (8) must be updated when $\mu_{m,k}$ changes—it is actually a parameter ${\tilde{\gamma }}_{m,k}$, depending on the time-instant *k* as well. In addition, reinitialization of (11) is recommended when significant changes are observed in the surrounding environment, i.e., different number of neighboring SUs or important differences in the estimated SNR conditions.

**Figure 2:**
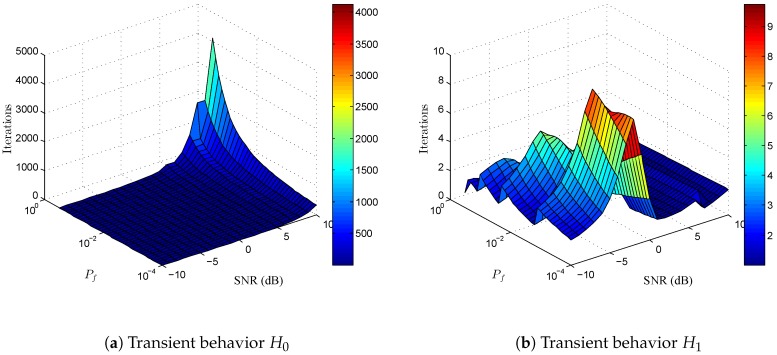
Number of iterations to decrease to $\frac{1}{e}$ of the initial value as a function of signal-to-noise ratio (SNR) and $P_{f}$ for hypotheses $H_{0}$ and $H_{1}$.

Additionally, the speed of convergence in the transient state can be approximated as $\frac{1}{\mu_{m,k}E\left[{\tilde{u}}_{m,k}^{2}\right]}$ iterations to decrease to 1/e of the initial value [35]. Since $E\left[{\tilde{u}}_{m,k}^{2}\right]$ are different for $H_{0}$ and $H_{1}$, transient states of both hypotheses would be different if the step-size were the same. Consequently, the overall probability of detection and the overall probability of false alarm will be degraded depending on the length of transient states compared with the length of steady state for the minimum lengths of hypotheses $H_{1}$ and $H_{0}$ given by the sampling rate of the adaptive ED algorithm. From Figure 1 and taking the convergence criterion in (10) into account, the variable step-size will be selected according to $\mu_{m,k}<E{\left[{\tilde{u}}_{m,k}^{2}\right]}_{H_{0}}^{-1}$ when SNR is negative. If we compute the 1/e-th transient periods, we see $\frac{1}{\mu_{m,k}E{\left[{\tilde{u}}_{m,k}^{2}\right]}_{H_{0}}}<\frac{1}{\mu_{m,k}E{\left[{\tilde{u}}_{m,k}^{2}\right]}_{H_{1}}}$, and, consequently, the overall probability of detection, $P_{d}$, could be more affected due to a longer transient state of $H_{1}$ when compared with the steady state. In contrast, when SNR conditions correspond to values in the red area depicted on the right-hand side of Figure 1, the step-size $\mu_{m,k}$ is upper-bounded by $E{\left[{\tilde{u}}_{m,k}^{2}\right]}_{H_{1}}^{-1}$ and transient time in $H_{0}$ will be higher than in $H_{1}$. As a result, the probability of false alarm will be degraded as compared to the target $P_{f}$. In Figure 2, we show the number of iterations that takes to decay 1/e-th of the initial value for $H_{0}$ and $H_{1}$ using a step-size computed as in (12), for the same parameters of SNR, $P_{f}$, and noise variance as in Figure 1. As mentioned before, one can observe that the 1/e-th transient period will be much greater for $H_{0}$ (≈[$10^{3},4\times 10^{3}$] iterations) than for $H_{1}$ (≈[1, 10] iterations) when SNR >5 dB and target $P_{f}>10^{-2}$. In contrast, when $P_{f}$ and SNR correspond to values in the blue area depicted on the right-hand side of Figure 1, transient states for both $H_{0}$ and $H_{1}$ become much smaller (≈[1, 100] iterations). As a result, since transient states must be negligible compared with steady states in order to achieve reasonable performance in terms of real $P_{f}$ and $P_{d}$, then the sampling rate associated with the signals feeding the adaptive algorithm must be chosen according to estimated SNR, target $P_{f}$, and the minimum average length between hypotheses $H_{0}$ and $H_{1}$. Otherwise, transient states are not negligible and overall performance is deteriorated.

### 3.3. Cooperative Detection Issues

When cooperative detection is performed at a given node *m*, the SUs belonging to the neighborhood $N_{m}$ (i.e., those users which can communicate with *m* using the control channel) must share some parameters with the *m*-th node. As shown in [24], the local noise variance and the test statistic of each neighboring user are the necessary parameters to share. Additionally, if weighted strategies are used, local SNRs could be also shared. In order to compute $\omega_{m,k}$, a series of additional operations must be carried out in order to get the inputs ${\tilde{u}}_{i,k}$ and desired signals ${\tilde{d}}_{i,k}$ from the local noise variances and the test statistics of each neighboring user in the neighborhood $N_{m}$. Reducing the number of those extra operations at each node necessarily calls for a more efficient sharing strategy consisting of sharing directly the inputs ${\tilde{u}}_{i,k}$ and desired signals ${\tilde{d}}_{i,k}$ instead of the local noise variance and the test statistics; this is indeed an efficient strategy since the proposed ${\tilde{u}}_{i,k}$ and ${\tilde{d}}_{i,k}$ are computed at each node anyway, i.e., there is no extra computational burden for the rest of neighboring users. On the other hand, due to the use of variable step-size algorithm, extra data must be shared in the proposed setup. In Table 1, we show the proposed shared data compared with the original algorithm in [24].

**Figure 3:**
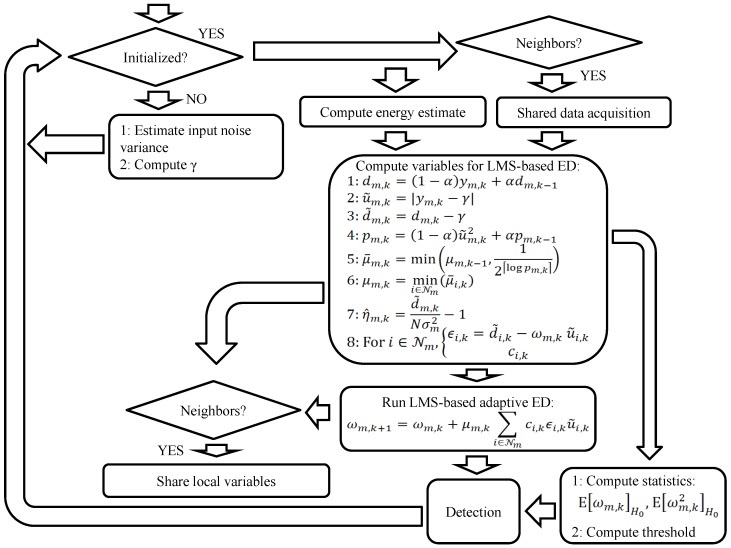
Flowchart of the adaptive variable-step-size-least-mean-squares (VSSLMS)-based energy detection (ED) algorithm.

Taking all the previous considerations into account, the flowchart of the practical ED algorithm is drawn in Figure 3. Firstly, the *m*-th SU must be initialized estimating the input noise variance $\sigma_{m}^{2}$, which will be later used to calculate $\gamma_{m}$ and statistics of $\omega_{m,k}$. Once the algorithm is initialized, the receiver can gather local and neighboring energy estimates, when cooperation is active, to perform the detection process. The computation of several parameters of the adaptive algorithm are conducted using $y_{i,k}\;i\in N_{m}$ and the initialization values. If the cooperation mode is active, the parameters shown in Table 1 are shared. Afterwards, the VSSLMS-based ED is executed and the detection threshold ${\tilde{\gamma }}_{m,k}$ is updated. Finally, the detection test is carried out.

## 4. Experimental SDR Testbench

**Figure 4:**
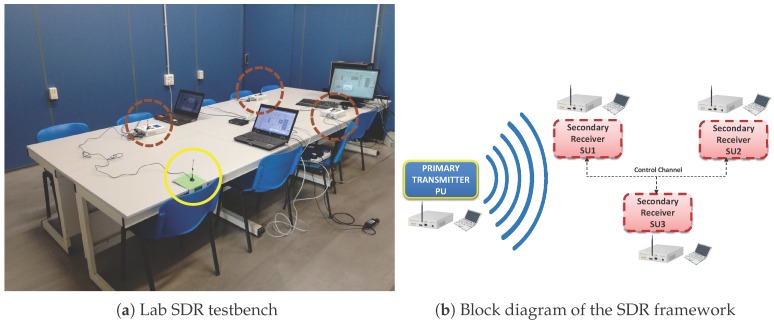
SDR framework formed by 1 primary user (PU) and 3 secondary users (SUs) which can work in stand-alone or cooperative modes. On the left-hand side, the PU antenna is marked by a yellow solid-line circle and SU antennas are marked by brown dashed-line circles.

**Figure 5:**
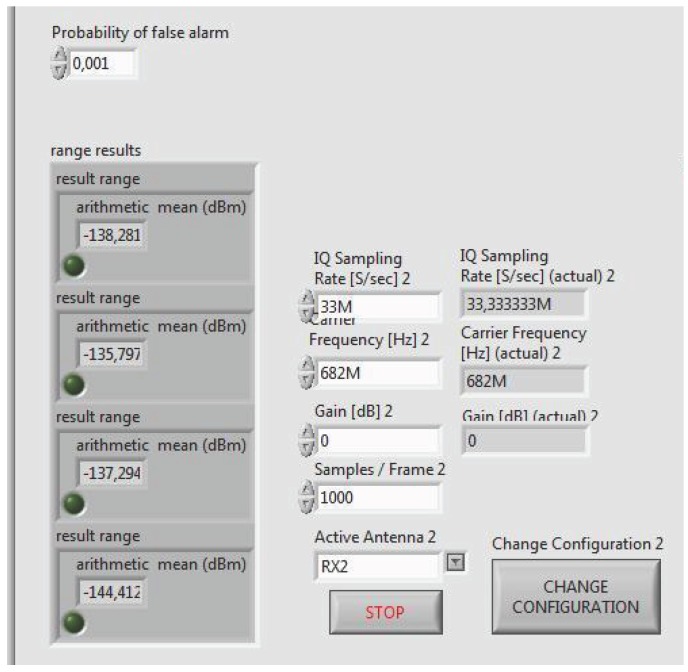
The depicted control panel allows for configuring $P_{f}$ and the parameters of receiving the data acquisition process. In addition, the panel shows on the left the computed detection thresholds for the selected channels.

**Figure 6:**
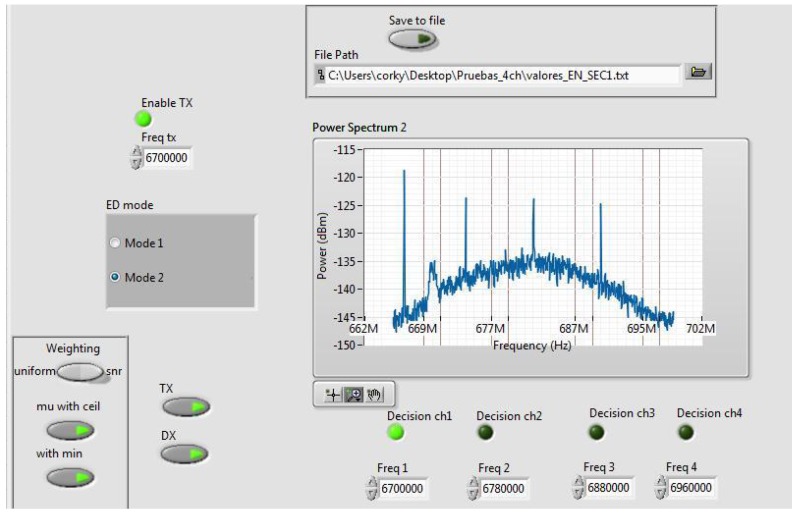
This control panel allows for configuring the variable step-size in (12), PU/SU roles, weighting strategy between uniform and SNR weighted, ED modes (single-node/cooperation), channel frequencies, and data saving. The power spectrum and the ED decisions for the selected channels are also depicted. In the snapshot, one can observe a PU transmission is detected in 670 MHz; cooperative mode (mode 2) with uniform weighting strategy is active; The Universal Software Radio Peripheral (USRP) device is working as PU transmitter from the transmission (TX) antenna (TX indicator on) and the SU receiver from the reception (RX) antenna (Detector (DX) indicator on); VSS is active (“ceil” and “min” flags on) and, finally, data is not saved.

The adaptive VSSLMS-based ED algorithm has been implemented by using three USRP devices (N-210 + Radio frequency (RF) daughterboard WBX, Ettus Research) and the LabVIEW software (version 14.0f1 (32 bits), National Instruments Corporation, Austin, TX, USA). The setup consists of 1 PU, which can transmit in a random manner maintaining each hypothesis state active during a minimum length, and 3 SUs, which can sense the environment in both single-node or cooperative modes. As shown in Figure 4, each transmitter or receiver antenna is connected to one USRP platform, which is controlled with LabVIEW software via Ethernet from one laptop or computer. All equipments are interconnected using Ethernet as a control channel in order to share data in cooperative mode. In Figure 5 and Figure 6, different parts of the LabVIEW control panel, used to configure the USRP device, are shown. From the panel in Figure 5, data acquisition physical layer (PHY) parameters can be controlled and target $P_{f}$ can be selected. USRPs receive IQ data with 33.3 MSamples/s centered at 682 MHz. IQ data stream is split into frames of 1000 time-domain samples to be converted to frequency domain. Furthermore, detection thresholds are also displayed for the selected channels. In Figure 6, we can set up the VSS in (12), PU/SU roles of the USRP, weighting strategy in cooperative mode, ED modes (single-node mode or cooperative mode), channel frequencies, and saving data. Additionally, the panel displays the power spectrum and the ED decisions for the selected channels.

The PU transmitter and SU receivers are operating in white spaces of the television (TV) band, i.e., free channels within the TV band (470–794 MHz). The receiver collects N=65 samples in the frequency domain from the selected channel bandwidth and computes the local energy estimates. The generated data traffic from PU consists of video transmission. The pre-defined probability of false alarm is fixed to 0.001. The hardware configuration and the parameter setting of the PHY layer of PU and SUs are summarized in Table 2. The selected channel is free of external RF interfering sources since they have been chosen from TV white spaces at that location. The minimum time length of each hypothesis is 10 s. In cooperative mode, the variables shown in Table 1 are shared with all users.

## 5. Results and Discussion

In this section, the proposed practical solutions in Section 3 are analyzed through lab measurements with the previously detailed SDR-based hardware implementation. Firstly, we assess in Section 5.1 how the transient-state affects the overall performance of the algorithm for different environments compared with the steady-state performance in terms of $P_{d}$ and $P_{f}$. Additionally, a comparison with the conventional ED method is also performed. In Section 5.2, the performances of the algorithm in real conditions are presented and compared with a theoretical benchmark for single-node and two-node cooperative ED.

### 5.1. Transient and Steady State Analysis

**Figure 7:**
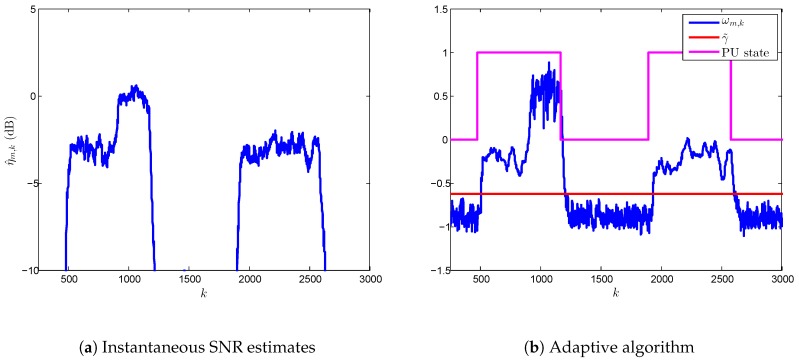
Behavior of the VSSLMS-based adaptive ED algorithm according to the PU states for single-node detection at SNRs in the range of [-4,0.5] dB.

**Figure 8:**
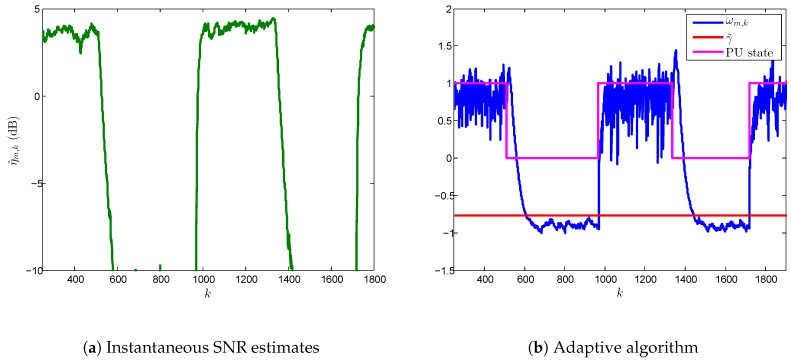
Behavior of the VSSLMS-based adaptive ED algorithm according to the PU states for single-node detection at SNRs in the range of [3,5] dB.

As we have seen previously, the length of transient states highly depends on the SNR at hypothesis $H_{1}$. We have analyzed this behavior in Figure 7 and Figure 8 using single-node detection in different environments where SNR varies in the ranges [-4,0.5] dB and [3,5] dB, respectively. The performance values in terms of $P_{d}$ and $P_{f}$ are shown in Table 3, where we have computed the overall detection performance and probability of false alarm, both in steady state, i.e., $P_{d}$ and $P_{f}$ after the adaptive ED algorithm converges. In addition, we have compared the results with conventional ED.

If we pay attention to Figure 7, we can see how the VSSLMS-based ED algorithm adapts their values to the time-varying nature of a receiving signal in real conditions. In addition, we observe that the transient state can be neglected compared with the steady state. In this sense, this effect is confirmed with results from Table 3 for the SNR range [-4,0.5] dB; transient state is similar in $H_{0}$ and $H_{1}$, and, consequently, the overall $P_{f}$ and $P_{d}$ are slightly increased and reduced, respectively. The ratio of false alarm is increased 0.035 and 0.038 compared with the traditional ED and steady-state $P_{f}$, respectively. In contrast, the $P_{d}$ performance is much higher than conventional ED despite the $P_{d}$ degradation induced by the transient state.

In Figure 8, we have measured time-varying PU signals in the interval [3,5] dB. On one hand, one can see that the transient state is much longer now than the one in Figure 7, thus inducing a degradation of $P_{d}$ and $P_{f}$ empirical values. As we have observed in Figure 2, a greater value of SNR leads to a major difference between the transient state lengths of the $H_{0}$ and $H_{1}$. In this context, that behavior can be observed in Figure 8b where transient state in $H_{0}$ is longer than $H_{1}$. Hence, the effect on the overall performance cannot be neglected compared with steady state, as shown in Table 3. This situation generates an important increase of $P_{f}$ (0.236) compared with the false alarm in steady-state (0.000) or in the conventional ED (0.013). It is worth noticing that both VSSLMS-based ED algorithm and conventional ED technique do not reach the target $P_{f}$ (i.e., 0.001), the former due to transient-state behavior and the latter due to the error at the noise variance estimation (The error in the noise variance estimation also affects the VSSLMS-based ED algorithm. However, its effect into the overall performance is negligible compared with that arisen from transient behavior.).

As a final remark, we can highlight that the practical proposal outperforms by far the conventional ED technique in low SNRs. However, despite the $P_{d}$ performance in SNRs above the interval [3,5] dB being better than conventional ED, their values are similar since traditional ED achieves good results in high SNRs. Furthermore, $P_{f}$ in adaptive ED is highly affected by transient states, providing an inefficient use of the idle channel. In this sense, a blended solution which could switch between adaptive and conventional ED for low and high SNRs, respectively, would be an interesting strategy to deal with all types of environments.

### 5.2. Detection Performance in Real Environments

In this subsection, the performance of the proposed practical VSSLMS algorithm is analyzed for single-node and cooperative ED detections in real propagation conditions. Conventional ED performance is also empirically measured. The experimental results are compared with theoretical simulation-based results employing MATLAB (Version R2012b, MathWorks, Natick, MA, USA). Theoretical results are computed using the same parameters as in the experimental setup for $5\times 10^{5}$ iterations.

**Figure 9:**
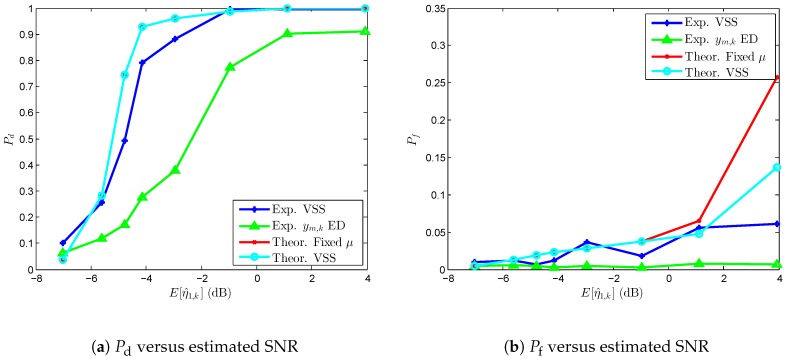
Performance comparison for different values of SNR for single-node detection.

Figure 9 shows $P_{d}$ and $P_{f}$ performances for different averaged measured levels of SNR in single-node detection. The results are compared with theoretical performance values of the LMS-based ED, selecting the step-size to satisfy the convergence criterion in (10), a theoretical VSSLMS-based ED, and the real performance of the conventional ED technique. The SNRs are averaged from the SNR estimates ${\hat{\eta }}_{m,k}$ computed as in (9). On one hand, one can see in Figure 9a that the values of $P_{d}$ performance of the proposed algorithm outperform the conventional ED technique for the measured range of averaged SNR. It is worth noting that the experimental VSSLMS-based ED provides $0.86<P_{d}$ for averaged SNR greater than -3 dB. If one pays attention to the probability of false alarm in Figure 9b, one can observe the negative effect of the transient states in the $P_{f}$ values as SNR increases compared with the conventional ED. On the other hand, when comparing the experimental results with theoretical values, we see a degradation on the $P_{d}$ values due to the time-varying environment in real conditions and possible estimation errors. In the case of $P_{f}$ performance, results are similar in the SNR interval [-7,1] dB and theoretical values are higher than experimental ones. This is due to the fact that, in real conditions, SNR variations modify transient states being longer and shorter according to the PU receiving signal fluctuations. However, in theoretical results, transient length is almost constant for the Monte Carlo simulation according to the pre-defined SNR. In addition, we can also see how the VSSLMS-based ED reduces $P_{f}$ compared with the LMS-based ED, while maintaining the same $P_{d}$ performance.

In Table 4, we analyze the performance of cooperative detection employing two SUs in cooperation mode with uniform weighting strategy (cf. [24] for more details) and we compare the performance results with the optimal linear cooperative strategy presented in [36]. The averaged SNR in SU 2 has been kept almost stable and the averaged SNR in SU 1 has been reduced using an attenuator at the input of the receiving antenna. The performance of the linear cooperative strategy has been computed using averaged SNR values of the SUs. One can observe that SU 2 with averaged SNR around -8.5 dB highly improves their performances compared with the single-node detection in Figure 9, i.e., $P_{d}<0.1$ for SNR <7. In contrast, SU 1 maintains similar $P_{d}$ and $P_{f}$ as single-node performances when averaged SNR >3 dB, whereas its $P_{d}$ values are degraded compared with single-node detection for $E\left[\eta_{1,k}\right]<3$ dB. From these results, we can conclude that cooperation among SUs is adequate when SNR levels are low, whereas SUs with high averaged SNR should share their local estimates with their neighbors with worse environment conditions but not to employ neighboring data in their local detection process proposed in Figure 3. The decision of cooperating could be performed comparing their local SNR estimates in (9) with a minimum SNR threshold before taking sharing data from other neighboring SUs. If we compare empirical and theoretical values, we can observe that experimental $P_{d}$ results are quite similar to the theoretical values for the SU 1’s SNR range [-4.21,0.49] dB. When the SU 1’s SNR is lower, theoretical $P_{d}$ becomes smaller. If we pay attention to the probability of false alarm, we can clearly observe the effect of the transient state in the theoretical performance. In contrast, empirical $P_{f}$ is almost stable for all measured averaged SNRs excepting the case of SU 1’s SNR =-1.77 dB, whose $P_{f}$ is abnormally high, i.e., $P_{f}=0.117$. Finally, if we compare the VSSLMS-based ED performance with the optimal linear cooperative ED presented in [36], we can observe that the proposed algorithm outperforms the linear ED strategy in terms of $P_{d}$. Nonetheless, the linear ED strategy presents a stable performance in terms of the desired $P_{f}$ maintaining its value as 0.001 for all cases.

## 6. Conclusions

In this paper, we have addressed the implementation issues of adaptive LMS-based ED previously proposed in [24] from a practical viewpoint. In this sense, a new practical VSSLMS-based ED proposal has been presented in order to deal with the convergence of the algorithm in real time-varying environments. Implementation guidelines of the new proposal for single-node and cooperative ED have been provided along with practical solutions to locally estimate the noise variance and SNR. The proposed VSSLMS-based ED has been implemented in an SDR-based hardware platform in order to assess its performance in real radio-propagation conditions. Experimental results show a good performance of the adaptive algorithm in terms of high $P_{d}$ ($P_{d}>0.9$) maintaining an adequate $P_{f}$ ($P_{f}∼0.05$) for an SNR range of [-4,1] dB in a single-node ED model. On the other hand, we have seen that an SU with low SNR (SNR ∼-8 dB) can achieve $P_{d}$ performance higher than 0.9 by sharing data with a neighboring SU with SNR ranges around [-2,0.5] dB. Moreover, we have observed that the practical proposal outperforms the optimal linear cooperative solution presented in [36] in terms of $P_{d}$ at a cost of a reduction of the opportunistic spectrum access when a channel is idle. A final remark is that the proposed adaptive ED solution is ideal for SNR <2 dB since the transient state of the algorithm degrades the overall performance if ED sampling rate is not increased. In this sense, blended solutions based on conventional ED in high SNRs and adaptive ED for low SNRs could be interesting for future research.

## Figures and Tables

**Table 1:** Shared parameters at each time slot *k* in cooperative detection.

	Theoretical LMS-Based ED [24]	Practical VSSLMS-Based ED
Mandatory	$\sigma_{m}^{2},\;y_{m,k}$	${\tilde{u}}_{m,k},\;{\tilde{d}}_{m,k},\;{\bar{\mu }}_{m,k}$
Optional	${\hat{\eta }}_{m,k}$	${\hat{\eta }}_{m,k}$

**Table 2:** Physical layer (PHY) parameters of primary (PU) and secondary users (SU).

User	Parameter	Value
PU & SU	Channel bandwidth	2 MHz
	Channel frequencies	670,678,688,696 MHz
PU	Modulation scheme	QPSK
	Symbol rate	1 MBaud
	Waveform	Single carrier
SU	IQ sampling rate	33 MHz
	Carrier frequency	682 MHz
	FFT	1000 samples
	ED sampling period	20 ms
	α in (5) and(12)	0.95

**Table 3:** Empirical $P_{d}$ and $P_{f}$ performances of variable-step-size-least-mean-squares (VSSLMS)-based and conventional energy detection (ED) with and without transient state performance taken into account.

SNR Range (dB)	States	Adaptive ED ($P_{d}/P_{f}$)	Conventional ED ($P_{d}/P_{f}$)
[-4,0.5]	Transient+Steady	0.955/0.038	0.508/0.005
[-4,0.5]	Steady	1/0.000		
[3,5]	Transient+Steady	0.993/0.236	0.904/0.013
[3,5]	Steady	1/0.000		

**Table 4:** Empirical and theoretical $P_{d}$ and $P_{f}$ performances for cooperative 2-node VSSLMS-based energy detection. Performance comparison with the optimal linear ED cooperation algorithm in [36].

$E\left[\eta_{1,k}\right]$ (dB)	$E\left[\eta_{2,k}\right]$ (dB)	Exp. VSSLMS ED	Theor. VSSLMS ED	Optimal Linear ED [36]
		($P_{d}/P_{f}$)	($P_{d}/P_{f}$)	($P_{d}/P_{f}$)
0.49	−16.20	0.984/0.044	0.995/0.039	0.966/0.001
−1.48	−8.12	0.968/0.020	0.971/0.032	0.754/0.001
−1.77	−7.84	0.954/0.113	0.965/0.032	0.706/0.001
−2.72	−8.56	0.801/0.017	0.890/0.027	0.517/0.001
−4.21	−8.53	0.468/0.023	0.431/0.021	0.274/0.001
−4.88	−8.64	0.339/0.023	0.269/0.018	0.197/0.001
−6.80	−8.32	0.187/0.015	0.090/0.009	0.082/0.001
−7.48	−8.66	0.103/0.038	0.051/0.006	0.058/0.001

## Abbreviations

The following abbreviations are used in this manuscript:

CR	Cognitive radio
SU	Secondary user
PU	Primary user
LTE	Long Term Evolution
LSA	Licensed Shared Access
REM	Radio-environment map
MCD	Measurement-capable device
ED	Energy detection
SNR	Signal-to-noise ratio
SDR	Software defined radio
USRP	Universal Software Radio Peripheral
LMS	Least mean squares
MAC	Medium access control
VSS	Variable step-size
RF	Radio frequency
PHY	Physical layer
TV	Television
